# A natural experiment identifies an impending ecological trap for a neotropical amphibian in response to extreme weather events

**DOI:** 10.1002/ece3.8848

**Published:** 2022-04-23

**Authors:** Morgan A. Clark, William M. Ota, Sierra J. Smith, Brett K. Muramoto, Summer Ngo, Gabriella E. Chan, Maxwell A. Kenyon, Matthew C. Sturtevant, Max G. Diamond, Gary M. Bucciarelli, Lee B. Kats

**Affiliations:** ^1^ 5262 Natural Science Division Pepperdine University Malibu California USA; ^2^ Department of Ecology and Evolutionary Biology UCLA Los Angeles California USA; ^3^ 8783 La Kretz Center for California Conservation Science Los Angeles California USA

**Keywords:** amphibians, Costa Rica, disturbance, natural experiment, trade offs, ultraviolet radiation

## Abstract

Extreme weather events are predicted to increase as a result of climate change, yet amphibian responses to extreme disturbance events remain understudied, especially in the Neotropics. Recently, an unprecedented windstorm within a protected Costa Rican rainforest opened large light gaps in sites where we have studied behavioral responses of diurnal strawberry poison frogs (*Oophaga pumilio*) to ultraviolet radiation for nearly two decades. Previous studies demonstrate that *O*. *pumilio* selects and defends perches where ultraviolet radiation (UV‐B) is relatively low, likely because of the lethal and sublethal effects of UV‐B. In this natural experiment, we quantified disturbance to *O*. *pumilio* habitat, surveyed for the presence of *O*. *pumilio* in both high‐disturbance and low‐disturbance areas of the forest, and assessed UV‐B levels and perch selection behavior in both disturbance levels. Fewer frogs were detected in high‐disturbance habitat than in low‐disturbance habitat. In general, frogs were found vocalizing at perches in both disturbance levels, and in both cases, in significantly lower UV‐B levels relative to ambient adjacent surroundings. However, frogs at perches in high‐disturbance areas were exposed to UV‐B levels nearly 10 times greater than males at perches in low‐disturbance areas. Thus, behavioral avoidance of UV‐B may not reduce the risks associated with elevated exposure under these novel conditions, and similarly, if future climate and human‐driven land‐use change lead to sustained analogous environments.

## Graphical abstract

Recently, an unprecedented windstorm within a protected Costa Rican rainforest opened large light gaps in sites where we have studied behavioral responses of diurnal strawberry poison frogs (*Oophaga pumilio*) to ultraviolet radiation for nearly two decades. In this experiment, we quantified disturbance to *O. pumilio* habitat, surveyed for the presence of *O. pumilio* in both high‐disturbance and low‐disturbance areas of the forest, and assessed UV‐B levels and perch selection behavior in both disturbance levels. In general, frogs were found vocalizing at perches in both disturbance levels, and in both cases, in significantly lower UV‐B levels relative to ambient adjacent surroundings, yet frogs at perches in high‐disturbance areas were exposed to UV‐B levels nearly 10 times greater than males at perches in low‐disturbance areas.

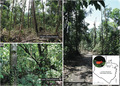

## 1 INTRODUCTION

Natural experiments offer a method to understand ecological outcomes that would otherwise be nearly impossible to experimentally execute due to practical or ethical limitations. Often the result of an unintended or catastrophic event, natural experiments provide a means to evaluate the outcomes of ecological disturbance (Diamond, 1983). Natural experiments offer researchers a way to study the factors that shape patterns and species behavior in response to a disturbance through observations of both short‐ and long‐term outcomes. For example, natural experiments have been leveraged to examine species response to wildfires, hurricanes, and forest canopy disturbance (Kerby & Kats, 1998; Schoener et al., 2001; Sousa, 1984; Stevens et al., 2015). Here, we investigate the ecological impacts of an unprecedented extreme climate event in the Neotropics that rapidly altered habitat and provided conditions for a natural experiment.

Amphibians, especially anurans, are considered bioindicators due to variable life‐history traits and sensitivity to mild environmental fluctuations (Blaustein & Wake, 1995). Many species demonstrate negative responses such as reduced larval survival, altered morphology, and behavioral abnormalities to rapidly altered environmental conditions associated with climate change, habitat destruction, disease, and invasive species (Alton & Franklin, 2017; Blaustein & Kiesecker, 2002) all of which have contributed to global amphibian population declines (Grant et al., 2016). Amphibian responses to major disturbances are less well studied in the Neotropics but may offer a better understanding of ecological outcomes that result from these events, especially in regions where populations have persisted after the emergence of infectious zoonotic pathogens (Wake, 2007).

**FIGURE 1:**
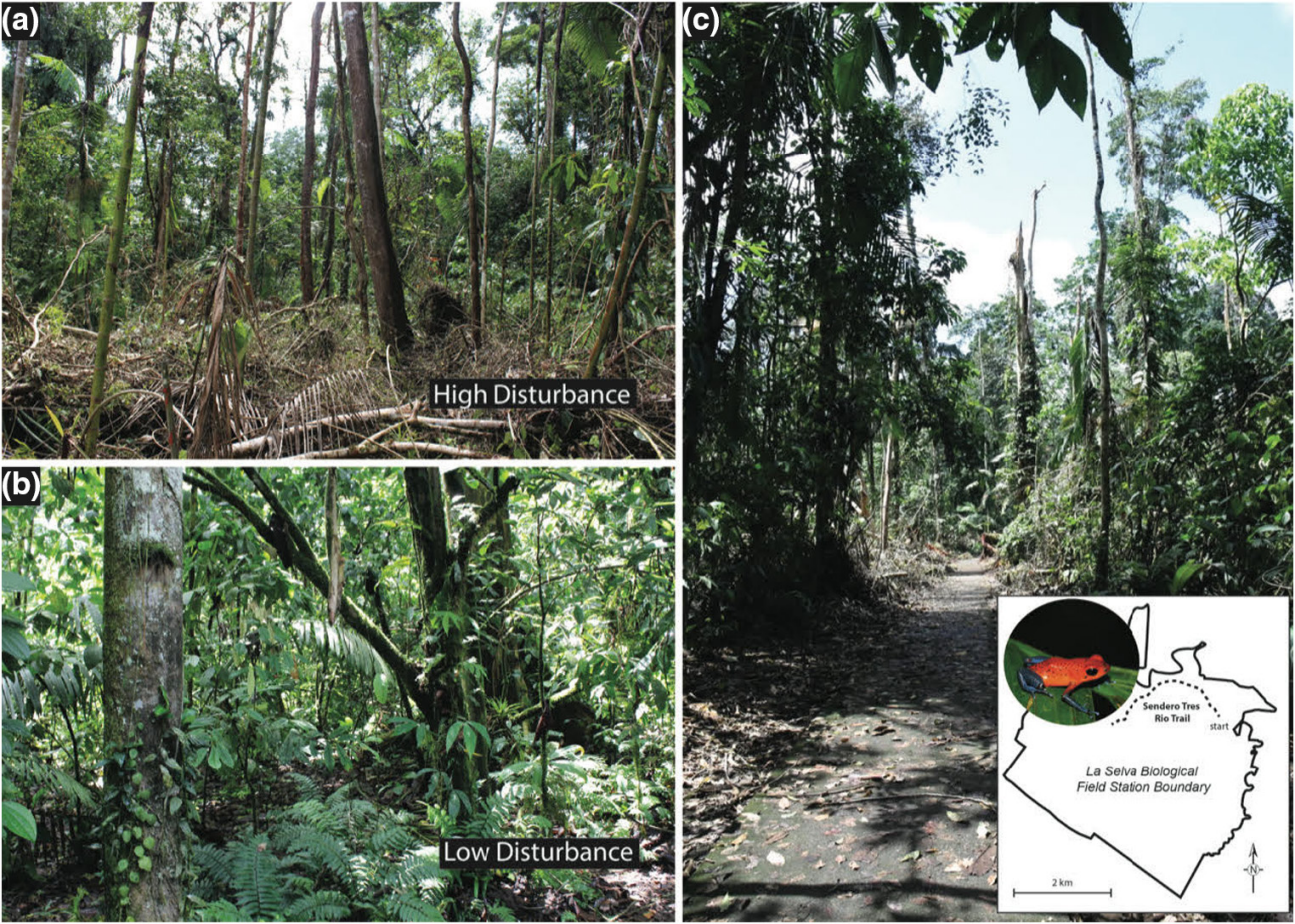
The impact of a novel extreme disturbance at a tropical field station. Photos show the impacts of a novel windstorm along study transects that we characterized as (a) high or (b) low‐ disturbance. (c) A map of the location of the study site at La Selva Biological Station (Heredia Province, Costa Rica, 10.43, −84.00) shows the focal species and main trail where transects were performed. Note that low‐disturbance areas are representative of the typical habitat that our focal species, the strawberry poison frog (*Oophaga pumilio*), inhabits

The frequency of extreme weather and climate events is predicted to increase throughout the neotropics and coupled with land‐use change, raises the need to better understand the ecological responses of forest‐dwelling species to these disturbances (Feng et al., 2013; Gonçalves et al., 2021; Ummenhofer & Meehl, 2017). On May 19, 2018, an unprecedented windstorm occurred within a protected rainforest at La Selva Biological Station (La Selva) (Puerto Viejo de Sarapiquí, Heredia, Costa Rica, 10.43, −84.00). This storm, described as an intense, acute, microburst, swept westward over La Selva, and was characterized by high wind speeds (5.2 m/s) that opened more than 600 isolated light gaps over 19.6 km of the forest (Rader et al., 2020) in sites where we have studied the behavioral responses of diurnal strawberry poison frogs (*Oophaga pumilio*) to ultraviolet radiation (UV‐B) for nearly two decades (Figure 1).

While various species traits across taxonomic groups could be of relevant interest following this storm, we focus here on the change to UV‐B conditions due to the biological relevance of ultraviolet radiation to our study species. In this study we quantified UV‐B exposure in the disturbed habitat and evaluated whether increases in UV‐B as a result of opened light gaps affected *O*. *pumilio* perch selection behavior. Ultraviolet radiation is known to negatively impact amphibian species through a range of lethal and sub‐lethal effects across life stages. For example, in developing amphibians, UV‐B exposure reduces amphibian larval survival, growth, locomotor performance, and induces developmental abnormalities. Malformations during development have been shown to alter behavior later in life, especially predator escape behaviors, which may have long‐term consequences for fitness and population recruitment (Alton & Franklin, 2017). Adult amphibians exposed to increased ultraviolet radiation have been observed to suffer increased skin damage and mortality, and may experience the physiological costs of DNA repair in response to UV‐B damage (Londero et al., 2019; Zavanella & Losa, 1981). Coupled with other stressors such as contaminants, pathogens, or environmental destruction, the effects of UV‐B exposure on amphibians may be magnified (Blaustein & Kiesecker, 2002).

The collective results of our previous research demonstrate that UV‐B strongly influences *O*. *pumilio* behavior. For example, UV‐B determines perch selection behavior for territorial *O*. *pumilio* (Han et al., 2007). Males select perches with UV‐B levels significantly below adjacent habitat and prefer to vocalize at perches where UV‐B is drastically reduced (~0.12 µW/cm^2^) presumably to limit the negative physiological or developmental impacts of increased exposure (Kats et al., 2012). Other neotropical amphibians have shown similar UV‐B avoidance behaviors, which suggests that behavioral adaptations to UV‐B are possibly widespread in diurnal neotropical amphibians (DeMarchi et al., 2018). As a result of upper and mid‐canopy strata loss due to the microburst (changes to habitat shown in Figure 1), we predicted that UV‐B levels on the forest floor would be higher in disturbed habitat, and subsequently, that these areas would experience drastic increases in UV‐B that likely would limit *O*. *pumilio* presence due to the previously observed negative male preference for high exposure perches (Han et al., 2007). Given the novelty of this storm event, we quantified disturbance within our study area, surveyed for and enumerated vocalizing male *O*. *pumilio* in both high and adjacent low‐disturbance forest, and measured associated UV‐B levels at perches.

## 2 MATERIALS AND METHODS

### 2.1 Field site and surveys

Data were collected at La Selva Biological Station, Costa Rica, approximately 17 days after an extreme windstorm created more than 600 isolated gaps in the forest (average gap size 180 m^2^, Rader et al., 2020). We surveyed along the Sendero Tres Rios trail in La Selva due to the historical prevalence of *O*. *pumilio* in this area and the extent to which the canopy was opened and trees felled by the windstorm. *Oophaga pumilio* is found year‐round in this location, and breeds continuously throughout the year. For these reasons plus accessibility, we chose to identify transects along the well‐established trail. A total of 20 survey transects along the trail were identified using trail markers. Survey transects were 20 m wide and 50 m long, centered on the trail at the transect midpoint. All data collection was approved by the Ministerio del Ambiente y Energía de Costa Rica.

To quantify disturbance in transects, we counted felled trees with a minimum of 10 cm diameter at breast height. Following the storm, some felled trees blocking the trail were cut through by research station staff in an effort to clear the trail. Any trees cut through to clear the trail were counted only once and all other felled trees were also, only counted once. Each transect was defined as high‐disturbance, where forest canopy and trees were downed, or low‐disturbance, where canopy was not visibly altered due to the storm (Figure 1).

To detect frogs, surveys were conducted on each transect on opposite sides of the trail. All data were collected between 0800 h and 1600 h for three consecutive days across the 20 transects, which were visited each day. Frogs were identified by vocalizations to ensure only males were counted because females do not call or utilize perches. After a vocalizing male was heard, we visually located the individual and measured UV‐B at the perch. Calling males unable to be visually located but identified within transects were counted, but no UV‐B measurement was taken.

UV‐B was measured following methods in Kats et al. (2012) using a PMA2100 Outdoor UV‐B meter (Solar Light Co., Philadelphia, PA, USA; sensor diameter: 24 mm; detectable range of wavelengths 280–370 nm). For each transect, ambient UV‐B was quantified first, and then UV‐B measurements at perch calling sites were taken. Ambient UV‐B was measured four times along a standardized route on the forest floor: (I) 12.5 m into the transect following the trail; (II) 25 m into the transect along the trail (midpoint); (III) 5 m into the forest directly perpendicular to the trail at the midpoint; and (IV) 37.5 m into the transect along the trail. Because transects were created along a trail with reduced canopy cover, ambient UV‐B levels within transects were calculated as the average of these four measurements, which better represents the average UV‐B of the habitat surrounding the entire transect. UV‐B at the frog perch was a single measurement, collected by placing the sensor on the perch where the male was visually observed. All measurements were taken parallel to the ground.

### 2.2 Statistical methods

We first quantified the number of felled trees in each transect and then evaluated whether the number of detected *O*. *pumilio* was affected by the number of felled trees. To do this, we fit a generalized linear model with a Poisson distribution that treated counts of *O*. *pumilio* as the response variable and number of felled trees as our predictor. Given that surveys were performed during three survey events that spanned three consecutive days, the same frogs could have been counted more than once. However, because each survey was performed unidirectionally, no frog could have been counted more than once during a survey event. Thus, to account for any potentially repeatedly counted frog between days, we included a random effect for each sampling event.

We then tested whether felled trees reduced canopy cover and thereby resulted in an increase of UV‐B levels where we detected vocalizing frogs. To do this, we regressed UV‐B levels measured at perches against the number of felled trees. We also used linear regression to test whether the UV‐B level at perches (*n* = 45) was significantly different from ambient (*n* = 45). The regression treated the two site types as a predictor and measured UV‐B as a continuous response variable. Finally, we used linear regression to detect whether the UV‐B levels where vocalizing frogs perched differed between high‐(*n* = 16) and low‐(*n* = 29) disturbance areas, and we then repeated the analysis to compare ambient levels in the two areas. All analyses were performed in *R* (*v*. 4.0.0) and all data met assumptions of normality.

## 3 RESULTS

**FIGURE 2:**
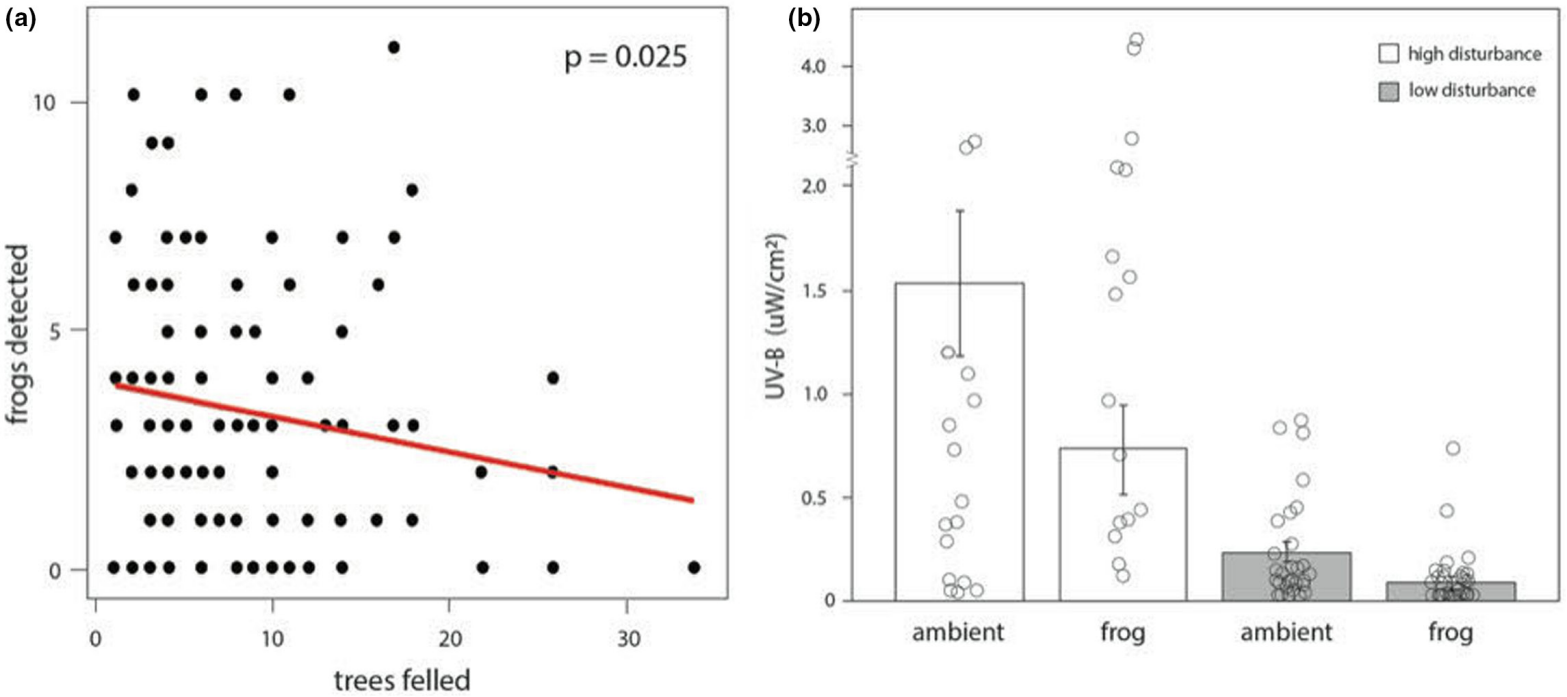
Impacts of the windstorm disturbance on the ecology of strawberry poison frog. (a) Significantly fewer frogs were detected in areas of the rainforest where more trees were felled and large light gaps were created along our transects. (b) In high‐disturbance areas, male frogs remained located in microhabitat with significantly lower UV‐B levels compared to ambient, but they still called from perches with significantly greater UV‐B levels relative to males in low‐ disturbance sections (GLM, *p* < .001, *z* = −4.06, *df* = 43)

We found that the number of detected *O*. *pumilio* was significantly affected by the number of felled trees (GLM, *p* = .003, *z* = −2.93, *df* = 110, Figure 2a). Our results indicated that the number of detected frogs was negatively correlated with the number of felled trees (β = −.025). Overall, the count of felled trees ranged from 0 to 34 within transects and averaged 8.98 (±1.1 SEM).

We determined from our linear regression results that the UV‐B levels at perches were affected by reduced canopy cover (GLM, *p* < .001, z = 4.72, *df* = 43). As the number of felled trees increased, there was a significant positive correlation with increased UV‐B levels at perches (β = .059). The UV‐B measured at perches ranged from 0.00 to 2.70 µW/cm^2^ ($\bar{x}$ = 0.30), whereas ambient UV‐B ranged from 0.00 to 4.40 µW/cm^2^ ($\bar{x}$ = 0.68; compared to ~15 µW/cm^2^ measured in areas with no canopy cover, see Han et al., 2007) and UV‐B levels significantly differed between perches and ambient (*p* < .001). Frogs in both high‐ and low‐disturbance areas of forest vocalized at perches with average UV‐B levels significantly lower than ambient UV‐B levels (frog: $\bar{x}$ = 0.29 µW/cm^2^ (±0.16 SEM); ambient: 0.67 µW/cm^2^ (±1.1 SEM)).

When we tested for differences between UV‐B levels at perches in high‐ and low‐disturbance areas of the forest, we found that vocalizing frogs in high‐disturbance areas of forest were calling at perches with significantly higher UV‐B levels ($\bar{x}$ = 0.74 µW/cm^2^) relative to the males vocalizing in low‐disturbance areas of forest ($\bar{x}$ = 0.08 µW/cm^2^) (GLM, *p* < .001, *z* = −4.06, *df* = 43, Figure 2b). In general, ambient UV‐B levels were significantly different between high‐ and low‐disturbance areas (GLM, *p* < .001, *z* = −4.93, *df* = 43, Figure 2b). The average UV‐B level at perches in high‐disturbance areas (0.74 µW/cm^2^, SD ± 0.89, range 0–2.7) was three times higher than the average ambient UV‐B in low‐disturbance areas (0.23 µW/cm^2^, SD ± 0.14, range 0–0.7).

## 4 DISCUSSION

An unusual and severe windstorm that cleared large swaths of secondary forest within a protected Costa Rican rainforest provided essential conditions for a natural experiment. This extreme event allowed us to test how local amphibians would behaviorally respond to a magnitude of increased UV‐B exposure in situ. Contrary to our predictions, we found that some frogs remained in large, opened light gaps and continued to exhibit perch calling behaviors consistent with previous studies (Han et al., 2007; Kats et al., 2012). However, the conditions in disturbed habitat generally exposed these frogs to novel UV‐B levels and significantly greater exposure at perch sites.

Previous studies demonstrate that UV‐B radiation generally influences amphibian perch selection behavior and that exposure is an important factor even at relatively low UV‐B levels when much of the forest canopy is intact and shielding amphibians from the majority of radiation (DeMarchi et al., 2018; Kats et al., 2012). In this study, *O*. *pumilio* were found vocalizing in disturbed and undisturbed habitat and in both habitat types located and called from perches with significantly lower UV‐B levels compared to the surrounding habitat. However, the males in high‐disturbance forest vocalized in UV‐B levels that were nearly an order of magnitude greater than the ambient levels of males in the low‐disturbance forest (Figure 2b). Thus, regardless of the disturbance, UV‐B remained a critical factor for male perch selection behavior. However, males in the disturbed habitat were exposed to UV‐B levels that exceeded what they would normally encounter and typically avoid under normal conditions, even without extreme events that rapidly shifted environmental conditions. Ultimately, male reliance on this behavior may be risky if the future environment resembles that of our natural experiment.

Vocalizing males in high‐disturbance forest relied on adaptive perch selection behaviors instead of abandoning large light gaps and shifting to adjacent habitat with 10‐fold reduced radiation. If males do not abandon disturbed forest and instead remain in habitat that exposes them to elevated UV‐B levels, then finding and selecting perches with the relatively lowest UV‐B levels would seem beneficial. However, we argue that UV‐B avoidance behavior in this context may be more costly to rely on instead of abandoning a site when conditions dramatically switch.

Consequently, UV‐B associated behaviors of *O*. *pumilio* appear double‐edged in the context of rapid environmental change: Despite males that resorted to UV‐B avoidance behaviors to locate and call from sites with the relatively lowest UV‐B levels, this tried‐and‐true behavior may not counteract the potential consequences of elevated exposure and in the longer term may be harmful, leading to steeper costs over time, especially as a greater frequency of storms and climate events is predicted to increase severe disturbances (Hulme & Viner, 1998). The impact of such events is of special concern in the tropics, as endemic species are predicted to respond most negatively to both long‐ and short‐term climate change‐associated disturbances, and the tropics continue to endure the sustained presses of increased temperature and deforestation (Chan et al., 2016; Corlett, 2012; Perez et al., 2016).

Numerous adaptive behaviors that species presently rely on may become ecological traps as a result of the increasing frequency of extreme climate events (Dale et al., 2001; Sih, 2013). In this system, potential avoidance of elevated UV‐B in these altered conditions may not be adequate to mitigate the imbalance between the benefits of remaining in disturbed habitat and the costs of increased exposure. A large body of research substantiates the negative impacts of UV‐B across amphibian life stages. For adult amphibians, harmful effects of UV‐B radiation may include increased mortality, irregular skin thickening and thinning, hyperplasia, thyroid system disruption, and DNA damage (Croteau et al., 2008; Licht & Grant, 1997; Londero et al., 2019). For diurnal frogs with parental care such as *O*. *pumilio*, sustained exposure to elevated UV‐B conditions may also negatively impact tadpole rearing behaviors, larval physiology, offspring survivorship, and parental care (Alton & Franklin, 2017; Dreher et al., 2017; Pröhl & Hödl, 1999; Romansic et al., 2009; Siddiqi et al., 2004; Weygoldt, 1980). While amphibians have evolved adaptations to withstand damage from ultraviolet radiation, including DNA repair mechanisms and protective pigmentation, these adaptations come with an energetic and physiological cost (Antwis & Browne, 2009; Londero et al., 2019).

Although there may be short‐term mate‐choice benefits related to visibility that drive males to risk greater UV‐B exposure (i.e., different UV‐B conditions affecting female perception of male frog coloration; see Dreher et al., 2017; Maan & Cummings, 2009; Summers et al., 1999), or potential benefits related to reduced intraspecific competition in disturbed sites, such a substantial increase and sustained exposure to drastically increased UV‐B at perches may impose too steep a fitness cost that negatively accrues at the individual and population level.

While *O*. *pumilio* consistently demonstrate avoidance of elevated UV‐B as a behavioral defense against increased ultraviolet radiation, this defense may not continue to be effective against drastic and rapidly increasing persistent levels of UV‐B exposure, like those seen after an abrupt disturbance to habitat (Blaustein & Belden, 2003). As such, under similar predicted conditions we speculate there may be rapid selection that modifies UV‐B avoidance, negates the benefits associated with potential trade‐offs, and shifts the behavioral ecology of diurnal amphibians.

Understanding how sudden and unpredictable disturbance events like this windstorm affect ecological responses is crucial to creating a framework for ecosystem response under the long‐term press of climate change and the punctuated pulses of associated extreme disturbance events. This press and pulse framework is necessary to form a comprehensive picture of future ecosystem stability and change, even for species and environments that are presently relatively undisturbed (Harris et al., 2018). By utilizing the naturally disturbed state of *O*. *pumilio* habitat, we were able to document a response to destructive pulse conditions in a previously untestable format. Our work demonstrates one immediate behavioral response of *O*. *pumilio* following an extreme, unprecedented storm, and sets the stage for further research by providing valuable insight into how extreme disturbance events alter ecological responses in a tropical system expected to experience future change. Going forward, researchers across ecosystems should begin to predict how species, populations, and communities might respond ecologically to environmental change induced by extreme climate and weather events, and for amphibians specifically, how we can use these events to assess long‐term stability of populations in the face of climate change‐associated disturbances, altered landscapes, and population declines.

## CONFLICT OF INTEREST

The corresponding author confirms on behalf of all authors that there have been no involvements that might raise the question of bias in the work reported or in the conclusions, implications, or opinions stated.

## AUTHOR CONTRIBUTIONS

**Morgan A. Clark:**Conceptualization (lead); Data curation (lead); Investigation (lead); Methodology (lead); Project administration (lead); Supervision (lead); Writing – original draft (lead); Writing – review & editing (lead). **William M. Ota:** Conceptualization (lead); Investigation (lead); Methodology (lead); Project administration (lead); Supervision (lead); Writing – review & editing (equal). **Sierra J. Smith:** Conceptualization (equal); Investigation (equal); Methodology (equal); Writing – review & editing (equal). **Brett K. Muramoto:** Conceptualization (equal); Investigation (equal); Methodology (equal); Writing – review & editing (equal). **Summer Ngo:** Conceptualization (equal); Investigation (equal); Methodology (equal); Writing – review & editing (equal). **Gabriella E. Chan:** Conceptualization (equal); Investigation (equal); Methodology (equal); Writing – review & editing (equal). **Maxwell A. Kenyon:** Conceptualization (equal); Investigation (equal); Methodology (equal); Writing – review & editing (equal). **Matthew C. Sturtevant:** Conceptualization (equal); Investigation (equal); Methodology (equal); Writing – review & editing (equal). **Max G. Diamond:** Conceptualization (equal); Investigation (equal); Methodology (equal); Writing – review & editing (equal). **Gary M. Bucciarelli:** Conceptualization (lead); Data curation (lead); Formal analysis (lead); Investigation (lead); Methodology (lead); Project administration (lead); Resources (lead); Software (lead); Supervision (lead); Validation (lead); Visualization (lead); Writing – review & editing (lead). **Lee B. Kats:** Conceptualization (lead); Data curation (lead); Funding acquisition (lead); Investigation (lead); Methodology (lead); Project administration (lead); Resources (lead); Software (lead); Supervision (lead); Validation (lead); Writing – review & editing (lead).

## ETHICAL APPROVAL

All data collection was approved by the Ministerio del Ambiente y Energía de Costa Rica.

### OPEN RESEARCH BADGES

This article has earned an Open Data Badge for making publicly available the digitally‐shareable data necessary to reproduce the reported results. The data is available at https://datadryad.org/stash/share/qJZRg45uQGbuYr_LzK2QgUFD1lpp6ZZm8Uc3BId_l4U.

## Data Availability

The dataset generated and analyzed during this study are available in the Dryad repository, https://datadryad.org/stash/share/qJZRg45uQGbuYr_LzK2QgUFD1lpp6ZZm8Uc3BId_l4U.
